# Food Insecurity Is Associated with Undernutrition but Not Overnutrition in Ecuadorian Women from Low-Income Urban Neighborhoods

**DOI:** 10.1155/2016/8149459

**Published:** 2016-03-23

**Authors:** M. Margaret Weigel, Rodrigo X. Armijos, Marcia Racines, William Cevallos

**Affiliations:** ^1^Department of Environmental Health, Indiana University Bloomington School of Public Health, Bloomington, IN 47405, USA; ^2^Programa Prometeo, Secretaría Nacional de Educación Superior, Ciencia, Tecnología e Innovación, Quito, Ecuador; ^3^Facultad de Ciencias Medicas, Universidad Central del Ecuador, Quito, Ecuador

## Abstract

Household food insecurity (HFI) is becoming an increasingly important issue in Latin America and other regions undergoing rapid urbanization and nutrition transition. The survey investigated the association of HFI with the nutritional status of 794 adult women living in households with children in low-income neighborhoods in Quito, Ecuador. Data were collected on sociodemographic characteristics, household food security status, and nutritional status indicators (dietary intake, anthropometry, and blood hemoglobin). Data were analyzed using multivariate methods. The findings identified revealed a high HFI prevalence (81%) among the urban households that was associated with lower* per capita* income and maternal education; long-term neighborhood residency appeared protective. HFI was associated with lower dietary quality and diversity and an increased likelihood of anemia and short stature but not increased high-calorie food intake or generalized or abdominal obesity. Although significant progress has been made in recent years, low dietary diversity, anemia, and growth stunting/short stature in the Ecuadorian maternal-child population continue to be major public health challenges. The study findings suggest that improving urban food security may help to improve these nutritional outcomes. They also underscore the need for food security policies and targeted interventions for urban households and systematic surveillance to assess their impact.

## 1. Introduction

It is estimated that, by 2050, 2.5 billion persons or two-thirds of the global population will be urbanites. Most of the increase is projected to occur in low- and middle-income populations [1, 2]. As of 2014, 80% of the population of Latin America and the Caribbean (LAC) was already residing in urban centers [2]. Meeting the food and nutrition security needs of urban households in the LAC region and elsewhere is a growing worldwide public health challenge. Food and nutrition security is defined as follows: “when all people at all times have physical, social and economic access to food, which is consumed in sufficient quantity and quality to meet their dietary needs and food preferences, and is supported by an environment of adequate sanitation, health services and care, allowing for a healthy and active life” [3]. Dietary diversity or the ability to access a wide variety of different foods is central to this concept. Diets that include both a wide variety of foods across as well as within major food groups are associated with better nutrition and health outcomes [4, 5].

Urban centers in low- and middle-income populations operate as cash-based economies where food and other household needs are obtained primarily through local market systems [6]. For this reason, the food security situation of urban households tends to be more tightly linked to their income and purchasing power compared to rural areas where it is usually more dependent on food availability linked to local production [7, 8]. Thus, having a steady and reliable source of cash income is critical since food costs alone may comprise as much as half or more of an urban household's monthly expenditures [9, 10]. Food security also is dependent on a consistent supply of foods in the urban marketplace at prices that are low enough for households to afford.

At the household level, food insecurity is characterized by the “limited or uncertain availability of nutritionally adequate and safe foods or the limited or uncertain ability to obtain foods by socially acceptable means” [11, 12]. Prior studies have documented that household food insecurity (HFI) which is acute or severe is associated with wasting and other indicators of severe undernutrition [12]. However, less severe episodic or cyclical HFI may also adversely impact the nutrition and health of women and other household members [12]. One of the ways in which it may do so is by affecting dietary quality and diversity. The findings from the few studies that have reported on the topic in LAC region countries, that is, Colombia and Trinidad and Tobago, suggest that mild-moderate episodic or cyclical food insecurity is associated with less frequent consumption of fruits, vegetables, dairy products, meat, and fish [13–16]. Such nutrient-dense foods are usually more costly pound-for-pound than lower quality items (e.g., white rice, noodles, bread, cassava, and potatoes).

Some recent studies also have implicated HFI in a higher prevalence of anemia [17, 18] and short stature in Ecuadorian [19] and Guatemalan women [18]. Anemia is a major public health issue in the LAC region because of high prevalence [20] and well-documented association with maternal mortality and morbidity, adverse maternal-perinatal outcomes, and diminished physical work capacity [20–22]. Likewise, short stature in adult women is an important public health concern due to its role in obstructed labor due to cephalopelvic disproportion and its association with reduced work capacity and economic productivity [23]. In addition, the offspring of short mothers are more likely to grow up stunted themselves and to produce stunted children who are at risk for future obesity [23, 24].

It has been hypothesized that HFI may promote weight gain and overweight/obesity in women through one or more complementary biobehavioral mechanisms involving diet, basal metabolism, stress hormones, and/or physical activity [12, 25–28]. However, the dietary and anthropometric data in support of the controversial “food insecurity-obesity hypothesis” is inconsistent. Studies conducted in high-income countries such as the US and Canada [12, 25, 28] and several LAC region countries including Colombia [13–15], Brazil [29, 30], Mexico [31], and Trinidad and Tobago [16] differ as to whether HFI promotes consumption of high-calorie foods, weight gain, and overweight/obesity in women and if so whether the direction of association is positive or negative.

Ecuador is one of a number of LAC region countries about which few HFI data have been published. Two-thirds of the residents of this rapidly urbanizing middle-income Andean country live in urban areas [32]. It has a well-developed agricultural sector, has an efficient food transportation system, and produces a wide variety of fresh fruits, vegetables, seafood, dairy products, meats, eggs, and other foods throughout the year. It also is a major food exporter and is food secure at the national level. Ecuador has made significant improvements in its economic and social situation during the past seven years. However, structural inequities still exist that decrease the access of impoverished households to sufficient nutritious food and other resources.

Data collected from the 2012 ENSANUT-ECU national health and nutrition survey strongly suggest that the Ecuadorian population is undergoing nutritional and epidemiologic transition. The survey findings indicate that the diet is becoming “westernized” with more refined grains, sugar, and fat [33, 34]. Unenriched white rice accounts for one-third of total dietary energy and African palm oil, a highly saturated cooking oil, accounts for 20% of lipids in the Ecuadorian diet. The survey results also indicate that energy-dense convenience, snack, and fast food intake by adolescents is common [34]. In addition, retail sales of ultraprocessed, sweetened beverages including soda pop, sports/energy drinks, and fruit juice/nectar drinks have steadily increased over the past 10 years [35]. The ENSANUT-ECU survey also reported low fruit and vegetable consumption among adults, averaging less than half of recommendations. Forty-five percent of Ecuadorian adults are reported to have low physical activity levels or are sedentary [33]. Similar to many other LAC countries, overweight and obesity often coexist with micronutrient malnutrition and stunting even within the same household or individual [33, 34]. Gastrointestinal, respiratory, vector-borne, and other infectious diseases remain common but obesity-related chronic diseases, that is, hypertension, heart disease, and diabetes, now rank as the top three causes of adult mortality [36].

Ecuadorian women and their children are particularly vulnerable to the double burden of undernutrition and overnutrition [19, 33, 34]. The prevalence of inadequate dietary micronutrient intake in women is high including vitamin A (88%), iron (78%), and zinc (11%) [33]. Anemia is the seventh leading cause of disability in the country affecting approximately 15% of women. Fifty-six percent also have zinc deficiency [33]. Approximately two-thirds of Ecuadorian women are overweight or obese [19, 33, 34] and one-sixth have short adult stature indicative of prior growth retardation during childhood or adolescence [19].

Quantitative data describing HFI and its correlates in the Ecuadorian population are scant. They include two small surveys conducted in rural farming communities in 2006 [37] and 2012 [38] and a 2014 analysis of household food insufficiency collected during the 2004 ENDEMAIN national reproductive health survey from 10,784 households with women aged 15–49 years [19]. The findings from these three surveys suggest that HFI seems to be relatively common and associated with lower food stores of meats, vegetables, legumes, and edible oils [37], reduced household expenditures on micronutrient-rich animal and plant foods [19], or lower intakes of animal protein foods [38]. The ENDEMAIN survey did not identify any association between HFI and excess body weight but did find that short stature was more prevalent among adult women [19].

It is unclear whether the limited findings from the aforementioned surveys are applicable to present day urban residents. Two of the three were conducted in rural farming households, both had very small sample sizes, that is, 52 households [37] and 113 households [38], and in one of these most of the participants were 60 years or older [38]. In addition, the data for two of the studies were collected around a decade ago when Ecuador was most likely in an earlier phase of the nutrition transition [19, 37]. Furthermore, although the 2012 study collected dietary intake data, it did not report on other nutritional indicators [38].

The major objective of the present study was to investigate the association of HFI with dietary, biochemical (blood hemoglobin levels), and anthropometric indicators of nutritional status in women from low-income households in a major urban center (Quito) in Ecuador. The working hypotheses were as follows: (1) due to their limited resources, food insecure women would be more likely than their food secure counterparts to report poorer quality and less diverse diets which would be lower in rich food sources of micronutrients and animal protein; (2) they also would be more likely to have anemia due to the poorer micronutrient content of their diets; (3) they would be more likely to show evidence of past stunting in the form of short adult stature caused by long-term economic inequities that promote food insecurity; and (4) they would more frequently consume high-calorie foods that promote excess body weight for reasons of lower cost, high-satiety characteristics, or other biobehavioral mechanisms.

## 2. Methods

### 2.1. Description of the Study Population

The study was conducted in Quito, the administrative and political capital of Ecuador which is situated in the Andes Mountains at average elevation of 2950 m above sea level (range: 500–4790 m) [39]. It is the second largest city in the country with 2.4 million residents and nearly one-fifth of the country's total population [40–42]. More than one-third (35%) of city residents are internal migrants, most of whom come from rural areas of highland provinces in northern and central Ecuador [43]. Quito also is one of the main destinations for political refugees and economic refugees from Colombia, Peru, Chile, Cuba, and Spain [44]. Official government figures indicate that, from 2010 to 2014, the proportion of Quito residents living in poverty ranged from 8.9% to 10.3%; extreme poverty affected 3–3.4% [45].

### 2.2. Study Site and Participants

The present work formed part of a larger investigation of the food, nutrition, and health issues of women and children from low-income urban neighborhoods. The survey study was conducted during the months of May-August in 2010, 2011, 2012, and 2014 in neighborhoods located in five Quito neighborhood sectors (Chillogallo, Cotocollao, El Dorado, El Camal, and Los Chillos). A nonprobabilistic sample of 794 adult female heads of households and their minor children aged 6–12 years were recruited through neighborhood public elementary schools and community health centers. The selection of mothers and their children in this age group was initially informed by our outreach work with local community partners from public elementary school parents and administrators and local medical professionals in the low-income Quito neighborhoods. They were concerned about the food, nutrition, and health of the children and their families. They were especially worried that many children on the* vespertino* (afternoon) school schedule were arriving at school without having eaten breakfast or lunch.

The face-to-face interviews with the mothers collected data on household food security status, sociodemographic characteristics, food intake, anthropometric indicators, and blood hemoglobin. To be eligible for study inclusion, prospective participants were required to be the mother of at least one school-aged minor child (6–12 years) who lived in the same household. They were also required to be a permanent resident of their current community and not to have any sensory or developmental conditions impeding their ability to understand and respond to questions. In cases where the mother was not living in the home due to divorce, death, or migration, the* de facto* female head of household (e.g., grandmother, aunt, and stepmother) living in the home participated. Study approval was obtained from institutional review boards at the University of Texas at El Paso and Biomedical Research Institute at the Central University of Ecuador. All of the women participants provided their informed written consent. Study participants received oral and written interpretations of their anthropometric, blood pressure, and laboratory screening results and as appropriate were given written referrals for follow-up health care.

### 2.3. Data Collection

#### 2.3.1. Household Food Security

The food security status of participant households was examined using the US Household Food Security Survey Module (HFSSM) Spanish version [46], adapted for use in Ecuador. The HFSSM was selected for use in the urban Quito group because it had been previously validated in rural Ecuadorian households [37] and when we first began our study, the Latin American and Caribbean Food Security Scale and other HFSSM-derived instruments were still undergoing development and testing and were not yet widely available. However, since the instrument had not been previously validated for urban households in Ecuador, we also decided to examine its internal validity, reliability, and other psychometric properties. Our findings which are detailed in the attached Supplemental File (see Supplementary Material available online at http://dx.doi.org/10.1155/2016/8149459) indicated that the performance of the language-adapted HFSSM was consistent with the theoretical framework of household food insecurity as a managed process and exhibited good validity and reliability.

The 18 questions contained in the instrument covered a spectrum of participant-reported household food access problems ranging from worry over running out of food to food deprivation in children. They included ten questions specific to adults or the household in general and eight others specific to minor children (< 18 years) living in the household [47]. Answers were classified as affirmative if the adult woman participant responded “yes,” “often,” “sometimes,” “almost every month,” and “some months but not every month” to the various questions. Affirmative responses to the 18 items were summed to construct the raw score which was used to classify households as food secure (score: 0–2) or as food insecure, that is, with low food security (score: 3–7) or very low food security (score: 8–18) [47]. Households classified with low food security reported reduced quality, variety, or desirability of diet with little or no indication of reduced food intake while those with very low food security reported multiple indications of disrupted eating patterns as well as reduced food intake [47].

#### 2.3.2. Participant and Household Characteristics

Data were collected during face-to-face interviews with the 794 adult female participants. The data collected on the characteristics of the participants and their households included age, education, marital status, pregnancy status, occupation, current employment status, monthly household income, birthplace, place of residence, neighborhood location, length of residence in current neighborhood, and family size.

### 2.4. Nutritional Status Indicators

#### 2.4.1. Self-Reported Dietary Quality

The participants were asked to rank the overall quality of their diet over the past 12-month period using a 5-point Likert scale where the possible responses ranged from excellent to poor [48]. Their responses were regrouped as fair/poor versus other for the statistical analysis.

#### 2.4.2. Food Frequency Questionnaire

A standard format food frequency questionnaire (FFQ) was used to collect data from participants on their usual intake of commonly consumed foods and beverages during the prior 12 months [49]. The specific foods and beverages contained in the FFQ were selected based on 24-hour dietary recall studies previously conducted by the authors in Quito and other populations in northern Ecuador and after additional consultation with local experts and key informants. The FFQ was piloted at an urban public health clinic in a low-income Quito neighborhood and then we refined the items as necessary. The FFQ food frequency categories ranged from never or less than once per month to 4–6 times per day. The response frequencies were converted into average weekly intakes for the analyses. The FFQ items were classified into major 11 groups: cereals, meats, eggs, milk/milk products, legumes/nuts/seeds, vegetables, fruits, fish/other seafood, white tubers/roots, sweets, and oils/fats (i.e., cooking oil, margarine, butter, and mayonnaise). These food group classifications followed the Food and Agricultural Organization (FAO) guidelines [50]. Because of interest in the association of food insecurity with energy-dense foods and obesity, we also added an additional 12th group consisting of high-calorie, high-fat, fried and salty snack and convenience foods obtained from street vendors, purchased from stores, or cooked at home. In addition, we created additional groups in order to examine the relationship of food security status with the intake of specific foods rich in retinol precursor carotenoids (i.e., dark leafy green vegetables, certain tubers, and fruits), retinol (i.e., egg yolks, fortified milk/milk products), and heme iron (i.e., flesh meats, fish/seafood) as well as animal protein foods (i.e., flesh meats, fish/seafood, eggs, milk, cheese, and yoghurt) [50].

### 2.5. Anthropometric Indicators

Data on body weight were obtained by weighing nonpregnant participants to the nearest kilogram without shoes or heavy clothing on a calibrated electronic balance (Detecto, Webb City, MO). The scale was recalibrated after each weighing. A portable stadiometer (Seca NA, Chino, CA) was used to measure standing height without shoes, hats, or other headwear. Complete sets of weight and standing height data were obtained from 703 participants to use in the calculation of body mass index (BMI), defined as weight (kg)/height (m^2^). Participants with BMIs of <18.5, 18.5–24.9, 25–29.9, and ≥ 30 were classified, respectively, with underweight, normal weight, overweight, and obesity [51]. We were able to obtain waist circumference measurements on 706 nonpregnant participants to assess abdominal obesity. Waist circumference was measured with a semiflexible anthropometric measuring tape (Seca NA, Chino, CA). Participants with waist circumferences measuring > 88 centimeters were classified with abdominal obesity following NHLBI/AHA recommendations [52]. Standing height measurements were used to classify short stature (<145 cms) in women participants aged ≥ 20 years [23]. We included both nonpregnant and pregnant women in these analyses. The rationale for restricting the analysis of short stature to women who were 20 years or older was to exclude adolescents who may not had yet achieved their final adult height [23].

### 2.6. Blood Hemoglobin

A sample of fingertip capillary blood was collected from participants for the purpose of measuring their blood hemoglobin concentrations. The blood samples were analyzed by the HemoCue® photometer (Model 201, HemoCue Inc., CA). Anemia manifested as low hemoglobin was used as a proxy indicator for iron deficiency in this study. Hemoglobin values were used to classify anemia according to established hemoglobin concentration cutoff-points [53]. The anemia classification categories for nonpregnant women were as follows: 11.0–11.9 g/dL (mild), 8.0–10.9 g/dL (moderate), and <8.0 g/dL (severe). The anemia classification categories for the 16 pregnant participants in the study were as follows: 10–10.9 g/dL (mild), 7.0–9.9 g/dL (moderate), and <7.0 g/dL (severe). The five Quito neighborhoods in which the participants lived ranged in altitude from 2453 meters (Los Chillos) to 3068 meters (Chillogallo) above sea level [54, 55]. The measured hemoglobin concentrations were adjusted for the altitude of participant residences following WHO [53] recommendations.

### 2.7. Data Analysis

The study data were analyzed using the IBM-SPSS statistical software, version 23 (IBM Corp.). The descriptive data are presented as number (%) or mean ± SD. Multinomial logistic regression was used to investigate the association of respondent and household characteristics identified in the initial analyses with the three HFI groups. This analytic method also was used to examine the association of HFI with overweight and obesity. Poisson regression with robust variance estimation was used to examine the association of HFI with iron-deficiency anemia, short adult stature, and abdominal obesity. The multivariate analyses adjusted for potential confounders including household* per capita* monthly income, maternal education, current Quito neighborhood residence (location) and length of residency, and data collection year. The data from the multinomial and Poisson regression analyses are presented as unadjusted and adjusted prevalence ratio (PR) estimates with 95% confidence intervals. Differences in the average number of different food group items consumed weekly by the study households and their average weekly consumption frequency of specific food groups were assessed using a general linear model adjusted for covariates in the multivariate analyses. *p* values < 0.05 were considered statistically significant.

## 3. Results

### 3.1. Participant and Household Characteristics

Table 1 displays the characteristics of the 794 women participants and their households.

### 3.2. Household Food Security

Eighty-one percent of the women participants reported that their households had experienced food insecurity sometime during the past 12 months. Forty-one percent (*n* = 325) reported low food security and 40% (*n* = 317) very low food insecurity.  Table 2 shows the results of the analyses indicating that monthly* per capita* income and years of participant formal education were inversely associated with HFI. In contrast, participants who had lived in the same Quito neighborhood for 50% or more of their lifetime were much less likely to live in a household with low or very low food security. After adjustment for the other model covariates, the contribution of participant age, marital status, and housewife occupation was no longer evident.

### 3.3. Dietary Indicators

#### 3.3.1. Self-Reported Dietary Quality

Twenty-eight percent (*n* = 91) and 59% (*n* = 186) of women participants living in low or very low food security households, respectively, ranked the quality of their diets over the previous 12 months as only fair to poor compared to 14.6% (*n* = 22) of those who were food secure. Participants living in household with low food security (adjusted PR = 1.94; 95% CI = 1.27, 2.96; *p* = 0.0001) or very low food security (adjusted PR = 4.03; 95% CI = 2.71, 5.99; *p* = 0.0001) were, respectively, nearly two and three times more likely than their food secure counterparts to report that their diet had been only fair to poor during the past 12 months.

#### 3.3.2. Dietary Diversity: Variation across and within Food Groups

Table 3 indicates that food insecure women, especially those from households with very low food insecurity affected with very low food security, reported generally less diverse diets during the previous 12-month period as measured by the amount of variation both across and within the 12 major food groups as well as within certain plant and animal food groups that are high in certain micronutrients and animal protein. However, they did not disproportionately consume a greater number of items from food groups considered to be energy-dense and micronutrient poor (e.g., sweets, fried and salty snack/convenience/fast foods).

As the adjusted analysis results displayed in Table 3 indicate, women from households with very low food security consumed a slightly lower average number of food groups compared to those from homes that were food secure or had low food security. However, this difference was small, that is, only two-tenths of a percent.

Table 3 also displays the findings of the adjusted analyses indicating that as compared to food secure women those with low and very low food insecurity had significantly reduced average intakes of different foods from the vegetable, fruit, meat, fish/seafood, milk/milk products, legumes/nuts/seeds, and white tubers/roots groups. The results also indicated that women with very low food security reported consuming fewer different items belonging to the sweets group compared to those who were food secure. In contrast, those from low food security households reported ingesting a slightly increased number of sweet foods. However, no statistically significant differences were identified in the mean number of different cereals, oils/fats, and fried and salty snack/convenience/fast food items reported as consumed by participants from the three household food security groups.

Food insecure women, especially those from household with very low food security, also appeared to have less diverse diets as indicated by the average number of different foods they reported consuming over the previous 12 months that were rich in retinol, retinol precursor carotenoids, heme iron, and animal protein.

#### 3.3.3. Food Consumption Frequency

Table 4 shows that participants from food insecure homes, especially those with very low food security, reported lower average weekly consumption of fruits, meats, fish/seafood, and milk/milk products compared to their food secure counterparts in the past 12 months. Those with very low food security also indicated that they had eaten vegetables less frequently than women living in households that were either food secure or having low food security. In contrast, no statistically significant differences were identified among participants from the three household food security groups regarding average weekly consumption of eggs, legumes/nuts/seeds, cereal, white tubers/roots, lipid/fats, sweets, and snack/convenience/fast foods. The adjusted analysis findings, displayed in Table 4, also indicate that participants from food insecure homes, particularly those with very low food security, reported less frequent weekly consumption of micronutrient-rich (i.e., retinol, carotenoids, and heme iron) and animal protein foods.

**Figure 1:**
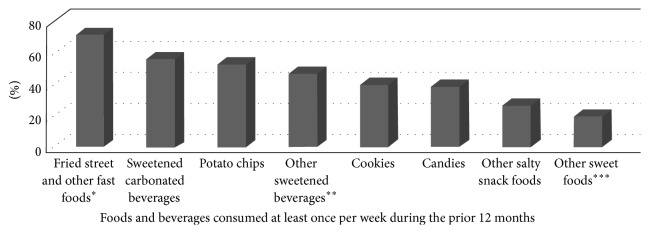
Reported weekly average consumption of sweet and fast/convenience/snack foods and beverages (*n* = 794). ^*∗*^Included items such as French fries, salchipapas (fried hot dog slices with French fries), fritada (fried pork pieces), empanadas, ripe plantains, and other fried foods. ^*∗∗*^Including items such as energy drinks, boxed fruit juice and nectar drinks, and other sugary processed beverages. ^*∗∗∗*^Included items such as donuts, cakes, meringues, jello, frozen confections, and other sugary foods.

No major differences were found in the proportion of women participants who reported that they had consumed sweet foods or fried and salty snack/convenience/fast foods at least once a week on average, during the past 12 months.  Figure 1 shows that approximately three-quarters of the women reported that they had regularly consumed fried/salty fast foods (e.g., French fries, hot dog slices, fritada (pork), hamburgers, empanadas, and ripe plantains) and more than one-half sugary carbonated beverages or potato chips at least once a week in the past year. The weekly consumption of the other energy-dense foods and beverages ranged from 19 to 40% (Figure 1). However, the adjusted analysis results did not identify any statistically significant differences in the consumption frequency of the food and beverage subgroups (data not shown).

### 3.4. Blood Hemoglobin and Anemia

Blood samples from 595 participants were available for the hemoglobin analyses. The altitude mean adjusted hemoglobin concentrations of women from households with low (12.7 ± 1.6 g/dL) or very low food security (12.3 ± 1.7 g/dL) were significantly reduced compared to those from food secure (13 ± 1.4 g/dL) households (*F* = 7.5; *p* = 0.001).

Slightly more than one-third (34.8%) were classified with anemia using the World Health Organization (WHO) criteria [56]. All of the anemia cases were classified as “mild” except for 13 (2.2%) who had moderate anemia and one who had severe anemia.  Table 5 displays the results of the adjusted analysis indicating that the prevalence of anemia among women living in households with more severe food insecurity, that is, very low food security, was nearly doubled compared to those who were food secure. However, low household food security, a milder form of food insecurity, was not associated with anemia. We were unable to analyze the association of household food security status with anemia severity. There were too few cases of moderate and severe anemia so the amount of available statistical contrast was too low to produce meaningful prevalence ratio estimates for the three anemia severity groups. However, as the WHO [56] has noted, “mild anemia” is a misleading term since iron deficiency is already advanced by the time anemia becomes detectable [56].

### 3.5. Anthropometric Indicators

No statistically significant differences were identified in the average BMIs of nonpregnant women from low (27.5 ± 13) and very low food security (28 ± 15) households compared to those living in food secure (27 ± 5) homes (*p* = 0.79). Based on their BMIs, 1% were classified as underweight and 38% as normal weight. In contrast, nearly two-thirds had BMI indicative of overweight (38%) or obesity (23%). More than half (53%) also had waist circumferences > 88 cm indicating abdominal obesity. However, as Table 6 shows, food insecure participants were not more likely than those who were food secure to be overweight or obese nor were they more likely to have abdominal obesity (Table 6).

Participant height averaged 1.51 ± 0.06 meters (range: 1.2–1.8 m). Fifteen percent of the women aged ≥ 20 years were classified with short adult stature (<1.45 m). Women from households with very low food security, but not less severe low food security, were more likely to show evidence of short adult stature although the strength of this association became marginal after adjustment for covariates.

## 4. Discussion

The present work is only one of three quantitative studies to investigate household-level food insecurity in Ecuador using an experienced-based, validated instrument and is the only one to specifically focus on urban women. In this study, HFI prevalence was high (81%) and associated with several undernutrition indicators suggesting micronutrient malnutrition in the urban Quito participants. However, it was not associated with a greater consumption of energy-dense foods and beverages nor with excess body weight or abdominal fat.

The prevalence of HFI in the urban Quito sample while slightly lower than figures quoted for two Ecuadorian rural highland farming groups [37, 38] was still substantial, affecting eight of every ten of the low-income households. It does not seem unreasonable to suggest that if HFI is this common in the capital city, it is likely that it exists at the same or even higher levels in other Ecuadorian urban centers where poverty rates are even greater [45].

As has been reported for Ecuador and other LAC region groups, as household income [15, 19, 57] and women's formal education declined [19, 29, 56], HFI increased. In contrast, long-term residence in the same Quito neighborhood appeared to be protective against HFI even after controlling for income and education. We surmise that this may be due to greater access of long-term residents to cheaper food outlets or better access to social safety net programs and social networks upon whom they can rely for food or cash gifts, loans, or trade. This factor should be explored in greater detail in future studies of urban HFI.

The finding that women living in food insecure homes, especially those with very low food security, had lower quality and less diverse diets is of particular concern since the consumption of a wide variety of good quality foods across and within major food groups is important for preconception, prenatal, and postnatal nutrition as well as overall health. Eating a diverse diet also ensures the presence of beneficial phytochemicals, promotes balance among nutrients that influence micronutrient absorption and utilization [20, 58], and reduces the risk for multiple micronutrient deficiencies [22].

The present findings also concur with emerging evidence linking more severe HFI to an increased prevalence of anemia in Mexican [17] and Guatemalan women [18]. In this study, the reduced levels of diversity both across and within food groups that characterized the diets of food insecure women appeared to be reflected in their higher prevalence of anemia based on the results of a* post hoc* analysis we conducted. Specifically, anemia was more common among women whose diets contained fewer different foods overall (*p* = 0.02) and those that had fewer foods containing heme iron (*p* = 0.03) and other animal and plant foods with nutrients that can act to enhance iron absorption and metabolism, that is, retinol (*p* = 0.003), carotenoids (*p* = 0.01), and ascorbic acid (fruits) (*p* = 0.003).

The results also concur with those of other recently published studies from Ecuador [19] and Guatemala [18] indicating that the prevalence of short adult stature was higher among women living in more severely food insecure households. This suggests that their families may have also suffered from HFI and so they were probably exposed to undernutrition during critical growth periods during their childhood or adolescence. These findings infer that interventions focused on improving household food security have the potential to also improve dietary diversity and micronutrient status which, in turn, could reduce the risk for nutritional anemias and stunting.

The data on the intake frequency of fast, snack, and convenience and other high-calorie foods and beverages reported by the adult women in our study are consistent with those also noted for adolescents in the recent ENSANUT-ECU survey [33]. However, the study data failed to support the hypothesis that HFI would be associated with the more frequent consumption of these types of foods and beverages. Our results concur with the small handful of studies published on this topic in LAC region countries [13–16]. The high (61%) prevalence of overweight/obesity identified among the participants is consistent with figures recently published for other Ecuadorian women [19, 33]. The study data, however, failed to support the hypothesis that HFI would be associated with excess body weight or abdominal fat in the urban Ecuadorian women. Although these results are similar to those noted for Ecuadorian women in the ENDEMAIN study [19] and for those in Colombia [13] and Trinidad and Tobago [16], they differ from what has been reported for women from Brazil [29, 30] and Mexico [31]. The reason for these discrepancies is not immediately evident but could be due to differences to where countries are in the nutrition transition, other population characteristics, or study methodology. In any case, the inconsistent findings for both energy-dense food intake and excess body weight mirror those previously reported for US and Canadian groups [12, 25, 28].

The present study has several potential limitations that should be considered when interpreting its findings. For example, the cross-sectional study design allows for inference but not establishment of temporal or causal effects. Since nonprobabilistic sampling was used that may not be representative of women living in other areas of Quito, those without children, or those with other characteristics. Another potential limitation concerns the study timing and length of data collection. This was due to logistical considerations related to the research team schedules and availability at the study sites during summer months only. In addition, since the assessment of food security status was limited to the past 12 months, it may not be necessarily representative of household situations over a longer period of time. Furthermore, since the development of anemia and overweight/obesity in women may take longer than 12 months covered in the food security survey instrument, we were forced to infer about past food security status. This was especially true for our assumption about short adult stature which usually results from undernutrition and other environmental insults (e.g., infection) that occur earlier in life during critical child and adolescent growth periods. For these reasons, it is suggested that future studies employ a longitudinal study design to simultaneously track HFI and nutritional indicators over time to confirm these relationships. It is also possible that, in some cases, the food security status of a household did not reflect that of a woman living in the same home as the findings from some studies suggest that the intrahousehold distribution of food and other resources may not always be equitable [59].

Ecuador has been making a concerted effort to improve the food, nutrition, and health of women, children, and other vulnerable subgroups. The new 2008 constitution guaranteed the rights of citizens to food and nutrition security, health, and other social protections. The Ecuadorian government, under its* Buen Vivir* National Development Plan, has identified specific nutrition and health priorities, goals, and action plans [60]. During the past decade, it substantially increased spending on its cash transfer, school feeding, nutrition education, maternal-child health, and other social safety net programs designed to reduce poverty and improve the quality of life [61]. Ecuador also has enacted new regulations on school meals and physical activity and recently implemented a mandatory requirement that all processed foods carry a “traffic light” label in traffic light color used to clearly indicate levels of fats, sugar, and salt in the item [62]. Ecuador also has banned the use of friendly animal characters, cartoon personalities, and celebrities for advertising foods and beverages with a high-fat, sugar, and salt content and is considering a new tax on “junk food” to reduce consumption and generate funding for antiobesity programs.

However, improving the food insecurity situation of Ecuadorian households requires that policy and program planners know where the most vulnerable households live are located and understand which factors promote and protect against it. We recommend that systematic surveillance be implemented in order to better target, monitor, and evaluate the outcomes of local and national food and nutrition security policies and programs for vulnerable Ecuadorian households. This requires the regular collection of up-to-date and disaggregated data.

We recommend using an experience-based instrument for surveillance purposes and research studies and other studies because these best capture the multiple quantitative and qualitative dimensions of food insecurity. The language-adapted experiential food security instrument used in this study exhibited good validity and reliability for the urban Quito households. However, investigators may also want to undertake validation of the Latin American and Caribbean Food Security Scale (*Escala Latinoamericana y Caribeña de Seguridad Alimentaria*) or the new FAO Food Insecurity Experience Scale (FIES) [63] for use in Ecuador since the use of harmonized, experience-based food insecurity measurement instruments would facilitate within- and between-population comparisons. In addition, the FIES instrument can be used for either household or individual level food security assessment [63].

## Supplementary Material

The Supplemental Material file describes the results of the Rasch Model analysis of the internal validity and other psychometric properties of the language-adapted Quito Household Food Security Survey.

## Figures and Tables

**Table 1:** Sociodemographic characteristics (*n* = 794).

Characteristics	Mean ± SD Number (%)
Monthly *per capita* household income ($US dollars)	110 ± 104
** **≤$50/month	173 (21.9)
** **$51–99/month	262 (33.1)
** **$100–149/month	206 (26.0)
** **≥$150/month	150 (19.0)
Education (years)	8.0 ± 3.8
** **<6 years	141 (17.8)
** **6–9 years	386 (48.6)
** **≥10 years	267 (33.6)
Age (years)	34 ± 10.6
** **<30 years	344 (43.3)
** **30–44 years	327 (41.2)
** **≥45 years	123 (15.5)
Marital status	
** **Legally married	235 (29.6)
** **Common law union	312 (39.3)
** **Single, never married	178 (22.4)
** **Separated/divorced/widowed	69 (8.7)
Occupation (full-time housewife)	417 (52.5)
Neighborhood location	
** **Chillogallo	162 (20.4)
** **Cotocollao	255 (32.1)
** **El Dorado	212 (26.7)
** **El Camal	102 (12.8)
** **Los Chillos	63 (7.9)
Quito birthplace	348 (43.8)
Long-term residency in current neighborhood (≥50% of lifetime)	483 (60.8)
Household size (number of members)	4.6 ± 1.6
** **2-3 members	174 (21.9)
** **4-5 members	447 (56.3)
** **≥6 members	173 (21.8)
Pregnancy status: currently pregnant	16 (2.0)

**Table 2:** Association of household and participant sociodemographic characteristics with food security status (*n* = 794).

	Household food security status			Low food security		Very low food security	
	Food secure (*n* = 152)	Low food security (*n* = 325)	Very low food security (*n* = 317)	Crude prevalence ratio (95% CI)	Adjusted prevalence ratio (95% CI)	Crude prevalence ratio (95% CI)	Adjusted prevalence ratio (95% CI)
Monthly *per capita* household income (US$)							
≤$50/month	12 (6.8)	56 (31.8)	108 (61.4)	4.37 (1, 13, 8.96)^1^	3.75 (1.75, 8.02)^2^	17.4 (8.29, 36.5)^1^	11.3 (5.01, 24.5)^1^
$51–99/month	39 (14.9)	109 (41.6)	114 (43.5)	2.62 (1.57, 4.36)^1^	2.26 (1.30, 3.92)^3^	5.65 (3.19, 10.0)^1^	4.06 (2.18, 7.55)^1^
$100–149/month	43 (20.9)	98 (47.6)	65 (31.6)	2.13 (1.29, 3.54)^1^	1.96 (1.16, 3.33)^4^	2.92 (1.63, 5.25)^1^	2.49 (1.34, 4.61)^3^
≥$150/month	58 (38.7)	62 (41.3)	30 (20.0)	1.00 (ref. cat.)	1.00 (ref. cat.)	1.00 (ref. cat.)	1.00 (ref. cat.)
Formal education (yrs)							
<6 years	23 (16.3)	42 (29.8)	76 (53.9)	1.22 (0.68, 2.18)	1.05 (0.56, 1.98)	4.37 (2.47, 2.74)^1^	2.69 (1.40, 5.15)^5^
6–9 years	47 (12.2)	160 (41.5)	179 (46.4)	2.27 (1.48, 3.48)^1^	1.70 (1.07, 2.71)^6^	5.04 (3.18, 7.98)^1^	3.32 (2.00, 5.49)^1^
≥10 years	82 (30.7)	123 (46.1)	62 (23.2)	1.00 (ref. cat.)	1.00 (ref. cat.)	1.00 (ref. cat.)	1.00 (ref. cat.)
Long-term residence in current neighborhood (≥50% of lifetime)							
Yes	116 (24.0)	191 (39.5)	176 (36.4)	0.44 (0.29, 0.68)^1^	0.47 (0.30, 0.73)^3^	0.39 (0.25, 0.60)^1^	0.43 (0.27, 0.69)^3^
No	36 (11.6)	134 (43.1)	141 (45.3)	1.00 (ref. cat.)	1.00 (ref. cat.)	1.00 (ref. cat.)	1.00 (ref. cat.)
Age (yrs)							
<30 years	76 (22.1)	157 (45.6)	111 (32.3)	1.00 (ref. cat.)	1.00 (ref. cat.)	1.00 (ref. cat.)	1.00 (ref. cat.)
30–44 years	56 (17.1)	136 (41.6)	135 (41.3)	1.18 (0.78, 1.78)	1.29 (0.81, 2.04)	1.65 (1.08, 2.53)^6^	1.65 (0.99, 3.74)
≥45 years	20 (16.3)	32 (26.0)	71 (57.7)	0.78 (0.42, 1.44)^7^	0.84 (0.43, 1.65)	2.43 (1.37, 4.32)^7^	1.93 (1.00, 2.71)
Full-time housewife occupation							
Yes	65 (15.6)	163 (39.1)	189 (45.3)^3^	1.35 (0.90, 1.99)	1.09 (0.71, 1.67)	1.98 (1.33, 2.93)^8^	1.28 (0.82, 2.02)
No	87 (23.1)	162 (43.0)	128 (34.0)	1.00 (ref. cat.)	1.00 (ref. cat.)	1.00 (ref. cat.)	1.00 (ref. cat.)
Marital status							
Legally married	44 (18.7)	82 (34.9)	109 (46.4)	0.74 (0.43, 0.27)	0.76 (0.41, 1.41)	0.58 (0.91, 2.73)	1.43 (0.73, 2.79)
Common law union	63 (20.2)	132 (42.3)	117 (37.5)	0.83 (0.51, 1.37)	0.81 (0.47, 1.38)	1.18 (0.70, 1.99)	1.27 (0.70, 2.30)
Single, never married	35 (19.7)	88 (49.4)	55 (30.9)	1.00 (ref. cat.)	1.00 (ref. cat.)	1.00 (ref. cat.)	1.00 (ref. cat.)
Separated/divorced/widowed	10 (14.5)	23 (33.3)	36 (52.2)	0.92 (0.40, 2.12)	0.84 (0.34, 2.06)	2.29 (1.01, 5.20)^9^	1.73 (0.69, 4.32)

Ref. cat. = reference category.

95% CI = 95% confidence interval.

Multivariate analyses adjusted for other model variables, neighborhood location, and data collection year.

^1^ *p* = 0.0001, ^2^ *p* = 0.001, ^3^ *p* = 0.004, ^4^ *p* = 0.012, ^5^ *p* = 0.003, ^6^ *p* = 0.021, ^7^ *p* = 0.002, ^8^ *p* = 0.005, and ^9^ *p* = 0.047.

**Table 3:** Variation across and within food groups during the past 12 months: comparison by household food security status (*n* = 794).

	Food secure (*n* = 152)		Low food security (*n* = 325)		Very low food security (*n* = 317)		ANOVA *p* value	ANCOVA *p* value^a^
	Mean ± SD	Adjusted mean^a^	Mean ± SD	Adjusted mean^a^	Mean ± SD	Adjusted mean^a^		
Variation across food groups								
Average number of food groups consumed	11.4 ± 0.8	11.4	11.4 ± 0.7	11.4	11.2 ± 1.0	11.2	0.001	0.003
Variation within food groups								
Average number of foods consumed within groups								
Vegetable group foods	8.5 ± 2.3	8.6	7.7 ± 2.6	7.7	7.3 ± 2.7	7.2	0.0001	0.0001
Fruit group foods	8.6 ± 3.0	8.6	7.9 ± 2.7	7.9	6.4 ± 3.4	6.4	0.0001	0.0001
Meat group foods	3.5 ± 1.3	3.4	3.2 ± 1.3	3.2	2.6 ± 1.3	2.7	0.0001	0.0001
Fish & seafood group foods	1.9 ± 1.0	1.9	1.7 ± 0.9	1.7	1.4 ± 0.8	1.4	0.0001	0.0001
Milk & milk products group foods	2.9 ± 0.8	2.9	2.8 + 0.8	2.8	2.2 ± 1.1	2.2	0.0001	0.0001
Legumes, nuts & seeds group foods	1.9 ± 0.8	1.9	1.7 ± 0.9	1.8	1.6 ± 0.9	1.5	0.0001	0.0001
Cereals group foods	5.4 ± 1.5	5.4	5.3 ± 1.4	5.3	5.1 ± 1.6	5.4	0.22	0.18
White tubers & roots group foods	4.5 ± 1.4	4.6	4.4 ± 1.5	4.4	4.1 ± 1.5	4.0	0.005	0.0001
Oils & fats group foods	2.2 ± 1.1	2.1	2.0 ± 1.1	2.0	1.8 ± 1.1	1.9	0.002	0.17
Sweets group foods	2.3 ± 1.4	2.2	2.5 ± 1.4	2.4	2.1 ± 1.3	2.1	0.0001	0.002
Eggs group^b^	—	—	—	—	—	—	—	—
Fried & salty snack group foods	1.7 ± 1.1	1.6	1.7 ± 1.1	1.7	1.6 ± 1.1	1.6	0.35	0.68
Total number of different foods consumed	43.8 ± 9.2	43.3	41.2 ± 9.0	41.2	36.4 ± 10.6	36.6	0.0001	0.0001
Number of micronutrient-rich foods consumed								
Retinol precursor carotenoid foods^c^	4.3 ± 1.6	4.3	3.9 ± 1.5	3.9	3.5 ± 1.6	3.5	0.0001	0.0001
Retinol foods^d^	3.9 ± 0.9	3.8	3.8 ± 0.9	3.8	3.2 ± 1.2	3.2	0.0001	0.0001
Heme iron foods^e^	5.4 ± 1.8	5.3	4.9 ± 1.7	4.8	4.0 ± 1.8	4.1	0.0001	0.0001
Number of animal protein foods consumed^f^	9.3 ± 2.3	9.1	8.6 ± 2.2	8.6	7.2 ± 2.5	7.3	0.0001	0.0001

^a^Analyses adjusted for household *per capita* income, age, education, length of residence in current neighborhood, neighborhood location, and data collection year.

^b^Eggs group contained only one food item (chicken eggs).

^c^Retinol precursor carotenoid key foods (dark leafy green vegetables, other vegetables, tubers, and vitamin A-rich fruits).

^d^Retinol key foods (egg yolks, milk/milk products).

^e^Heme iron key foods (flesh meats, fish/seafood).

^f^Animal protein food (flesh meats, fish/seafood, eggs, and milk/milk products).

**Table 4:** Average weekly food consumption frequency during the past 12 months: compared by household food security status (*n* = 794).

	Food secure (*n* = 152)		Low food security (*n* = 325)		Very low food security (*n* = 317)		ANOVA *p* value	ANCOVA *p* value^a^
	Mean ± SD	Adjusted mean^a^	Mean ± SD	Adjusted mean^a^	Mean ± SD	Adjusted mean^a^		
Weekly food group intake frequency								
Vegetable group foods	2.4 ± 1.5	2.4	2.4 ± 1.4	2.4	2.1 ± 1.5	2.1	0.51	0.011
Fruit group foods	2.2 ± 1.2	2.2	1.9 ± 1.2	2.0	1.4 ± 1.2	1.4	0.0001	0.0001
Meat group foods	2.0 ± 1.2	1.9	1.8 ± 1.2	1.8	1.3 ± 1.1	1.4	0.0001	0.0001
Fish & seafood group foods	1.4 ± 1.3	1.4	1.3 ± 1.2	1.3	1.0 ± 0.9	1.0	0.0001	0.0001
Milk & milk products group foods	3.7 ± 1.9	3.7	3.4 ± 2.0	3.4	2.3 ± 2.0	2.3	0.0001	0.0001
Legumes, nuts & seeds group foods	1.8 ± 1.2	1.8	1.7 ± 1.5	1.7	1.6 ± 1.7	1.6	0.64	0.28
Cereals group foods	3.3 ± 1.5	3.2	3.5 ± 1.8	3.5	3.3 ± 1.6	3.3	0.0001	0.17
White tubers & roots group foods	2.5 ± 1.4	2.5	2.6 ± 1.6	2.6	2.5 ± 1.5	2.5	0.53	0.27
Oils & fats group foods	2.6 ± 1.8	2.6	2.4 ± 1.7	2.4	2.3 ± 1.9	2.3	0.29	0.33
Sweets group foods	1.5 ± 1.6	1.5	1.6 ± 1.6	1.6	1.4 ± 1.6	1.4	0.16	0.36
Eggs group	4.6 ± 3.0	4.6	4.7 ± 2.4	4.6	4.4 ± 3.1	4.5	0.47	0.85
Fried & salty snack & convenience group foods	1.4 ± 1.4	1.4	1.6 ± 1.7	1.6	1.5 ± 1.6	1.5	0.35	0.41
Micronutrient-rich foods								
Retinol precursor carotenoid foods^b^	1.5 ±1.1	1.6	1.4 ± 1.1	1.4	1.1 ± 1.0	1.0	0.0001	0.0001
Retinol foods^c^	3.9 ± 1.8	3.8	3.6 ± 1.8	3.6	2.7 ± 1.8	2.7	0.0001	0.0001
Heme iron foods^d^	1.8 ± 1.1	1.7	1.6 ± 1.0	1.6	1.2 ± 1.0	1.2	0.0001	0.0001
Animal protein foods^e^	2.5 ± 1.1	2.5	2.3 ± 1.1	2.3	1.7 ± 1.1	1.8	0.0001	0.0001

^a^Analyses adjusted for household *per capita* income, age, education, length of residence in current neighborhood, neighborhood location, and data collection year.

^b^Retinol precursor carotenoid key foods (dark leafy green vegetables, other vegetables, tubers, and vitamin A-rich fruits).

^c^Retinol key foods (egg yolks, milk/milk products).

^d^Heme iron key foods (flesh meats, fish/seafood).

^e^High quality animal protein food (flesh meats, fish/seafood, eggs, and milk/milk products).

**Table 5:** Association of household food sufficiency status with participant anemia status (*n* = 595).

	Household food security status			Low food security		Very low food security	
	Food secure (*n* = 115)	Low food security (*n* = 239)	Very low food security (*n* = 241)	Unadjusted prevalence ratio (95% CI)	Adjusted prevalence ratio (95% CI)	Unadjusted prevalence ratio (95% CI)	Adjusted prevalence ratio (95% CI)
	*n* (%)	*n* (%)	*n* (%)				
							
Anemia	25 (21.7)	81 (33.9)	101 (41.9)	1.85 (1.10, 3.10)^1^	1.53 (0.98, 2.40)	2.60 (1.60, 4.30)^2^	1.94 (1.24, 3.03)^3^

Blood hemoglobin classified using WHO [54, 55] reference standards for anemia, adjusted for altitude, age, and reproductive status.

95% CI = 95% confidence interval.

Analyses adjusted for household *per capita* income, education, length of residence in current neighborhood, neighborhood location, and data collection year.

^1^ *p* = 0.02; ^2^ *p* = 0.0001; ^3^ *p* = 0.004.

**Table 6:** Comparison of household food security status with anthropometric indicators.

	Food secure *n* (%)	Low food security *n* (%)	Very low food security *n* (%)	Low food security		Very low food security	
				Unadjusted prevalence ratio (95% CI)	Adjusted prevalence ratio (95% CI)^a^	Unadjusted prevalence ratio (95% CI)	Adjusted prevalence ratio (95% CI)^a^
Generalized obesity (*n* = 703)	(*n* = 130)	(*n* = 277)	(*n* = 296)				
Overweight (BMI 25–29.9)	53 (40.8)	119 (43.0)	123 (41.6)	0.95 (0.58, 1.56)	0.90 (0.54, 1.49)	0.93 (0.57, 1.52)	0.80 (0.48, 1.34)
Obesity (BMI ≥ 30)	39 (30.0)	68 (24.5)	78 (26.4)	0.74 (0.43, 1.27)	0.70 (0.39, 1.22)	0.80 (0.47, 1.37)	0.61 (0.34, 1.09)
Abdominal obesity (*n* = 706)	(*n* = 138)	(*n* = 280)	(*n* = 288)				
Waist circumference > 88 cms	70 (50.7)	147 (52.5)	158 (54.9)	1.04 (0.78, 1.38)	1.03 (0.77, 1.37)	1.08 (0.82, 1.43)	1.00 (0.74, 1.33)
Short adult stature (*n* = 744)	(*n* = 143)	(*n* = 298)	(*n* = 303)				
Standing height < 143 cms	12 (8.4)	39 (13.1)	61 (20.1)	1.56 (0.82, 2.98)	1.34 (0.73, 2.66)	2.40 (1.29, 4.46)^1^	1.89 (1.01, 3.56)^2^

95% CI = 95% confidence interval.

^a^Analyses adjusted for household *per capita* income, age, education, length of residence in neighborhood, neighborhood location, and data collection year.

^1^ *p* = 0.006; ^2^ *p* = 0.049.
