# Supplementary material for: Food Insecurity Is Associated with Undernutrition but Not Overnutrition in Ecuadorian Women from Low-Income Urban Neighborhoods
Source: J Environ Public Health. 2016 Mar 23;2016:8149459. doi: 10.1155/2016/8149459 (PMC4821969; doi:10.1155/2016/8149459)
Supplement: Supplementary file 1 — The Supplemental Material file describes the results of the Rasch Model analysis of the internal validity and other psychometric properties of the language-adapted Quito Household Food Security Survey. [file 8149459.f1.docx]

**Supplemental File**

The Rasch Model, a one-parameter logistic item response model was used to examine

the internal validity and other psychometric attributes of the language-adapted Quito Household Food Security Survey (Quito HFSSM). The Rasch Model has been extensively used in the development and assessment of the original U.S. Household Food Security Survey Model (HFSSM) and its language-adapted versions and derivative instruments [1,2]. The basic assumptions of the Rasch Model are that the items in an instrument are unidimensional, they measure the same construct, i.e., household food insecurity (HFI), and are conditionally independent [1,3].

Participant responses to each of the 18 Quito HFSSM items were coded as either one (affirmative) or zero (negative). These were then fitted into the Rasch Model using WINSTEPS software, version 3.91 (WINSTEPS, Beaverton, OR). Model assumptions about unidimensionality and same construct measurement were assessed using “fit” statistics. Specifically, item infit and outfit statistics were calculated to ascertain how well responses to survey items fit the Rasch Model. The WINSTEPS program calculated the fit statistics by squaring the difference between the modeled and actual responses and averaging that sum [4]. The results were then squared to produce to approximate a unit normal or z-distribution.

Item infit statistics compare item strength and latent trait consistency, in this case, HFI, to the average of all items in the survey. This information-weighted sum gives more value to on-target observation. An item-infit score of one suggests a response that was a perfect fit to the model. Item misfit is suggested by higher values, i.e., scores above one, which signals weaker associations. In contrast, scores below one indicates that fewer persons answered affirmatively to an item than would be expected and suggests possible item redundancy. The recommended range of scores is 0.8 to 1.2 [5]. Item outfit is the conventional averaged sum of squared standardized residuals. These are an outlier-sensitive fit statistic and since they are unweighted, are easily influenced by just a few unexpected or extreme responses. For this reason, investigators rely more on item infit statistics because these weight the response of households closest to the item value [6].

The independence of the items in the Quito HFSSM scale was assessed using measure values which indicate the relative severity of each survey item in relation to the actual food security status of participant households. The Rasch Model assumes that the more food insecure the household is the more likely it is that the answer will be affirmative to each question and vice-versa. As recommended, households with extreme responses, i.e., those that affirm or none of the questions, were excluded from the scaling analysis since they do not provide any information on item relative severity [7]. The calibrations or severity parameter estimates generated by WINSTEPS were estimated on a logistic scale. These were then rescaled to positive values by adjusting the mean to a value of 7.0 [3].

The Quito HFSSM items were plotted with corresponding items from the U.S. Current Population Survey to compare the extent to which the two instruments measure the same basic phenomenon [3]. To facilitate this comparison, a linear transformation was used to adjust the Quito HFSSM item severity values in order to equate that of item scores to the equivalent U.S. CPS food insecurity items [3].

After the basic Rasch Model assumptions were met, Differential Item Functioning (DIF) was used to make comparisons across several different groups. This type of analysis permits comparison of the relative severity of each item between two groups in order to determine whether or not responses to the items are being influenced by group membership. A substantial DIF contrast score (> 0.5 logit units) infers that the response probabilities are not being completely explained by the latent trait of interest but rather by other variables that are influencing the response [4]. In this study, DIF was used to examine the influence of adult female education (< 6 vs. > 6 years), low income (household *per capita* income < $50/month vs. > $50/month), adult female occupation (full-time housewife vs. other), and neighborhood residency stability (< 50% vs. > 50% of lifetime living in same neighborhood).

Finally, the predictive ability or external validity of the Quito HFSSM was assessed by investigating its association with factors documented or expected to be causally related to HFI or its consequences. For these analyses, we examined the ability of the Quito HFSSM to classify HFI categories based on well-documented food access indicators such as income and education and dietary outcomes previously linked with household food security status.

The results of the internal validity and other analyses are displayed in the two tables and the figure. Table 1 shows the proportion of affirmative responses to each of the Quito HFSSM item. These are ordered by the level of severity of food insecurity. Conceptually less severe questions (e.g., worried would run out of food before being able to buy more) showed a higher proportion of positive responses than those which were conceptually more severe in nature (e.g., adults or children go without food for a whole day) [1,2,8]. The reliability of the Quito HFSSM, as assessed by Cronbach’s alpha, was 0.89.

Table 1 also shows the infit statistics for individual Quito HFSSM items. As indicated, these ranged from 0.85-1.25 and indicated that there were no substantial deviations from expectations for individual items. Although the infit value for the first survey question (HH2) was slightly elevated (1.25), it was still within the recommended range [Bickell 2000]. The overall mean item-infit score was 0.97 with a standardized score of −0.5 suggesting a solid unidimensional construct.

The relative item severity values (calibrations) for the scale items are shown in Table 1. Congruent with Rasch Model expectations, as the conceptual severity of the items increased, the relative severity values also increased in a monotonic fashion. These ranged from the survey item with the lowest severity measure, “worried about food running out” to the item with the highest severity measure, “child did not eat for a whole day”. Figure 1 displays the comparisons of the item severity values for the Quito HFSSM with the U.S. CPS. As shown, the Quito HFSSM items are in the same general order of severity as the U.S. CPS. The two scales had similar severity values although some of the Quito HFSSM adult items were higher and some of the child items were lower.

Table 2 displays the results of the DIF contrast analyses which examined the potential influence of group membership on the relative severity values of the Quito HFSSM items. The overall DIF analysis results confirmed that, with very few exceptions, the response probabilities appeared to be well-explained by the latent trait rather than by participant characteristics. Differences in participant responses were noted for only three items, one with education and two with residential length.

The predictive ability of the Quito HFSSM to categorize food insecurity was substantiated based on the income and education characteristics of households. In households where the mean monthly *per capita* income was < $50, $51-99, $100-149, and > $150 per month, the respective prevalence of severe HFI (i.e., very low food security) was 61%, 44%, 32%, and 20%. The adjusted prevalence of severe food insecurity was 17 times higher among households with a mean monthly *per capita* income of < $50 compared to those where it was > $150/month (see Table 2 in main paper). Likewise, when the educational level of the mother/other adult female head of household was < 6 years, 6-9 years and > 10 years, the prevalence of severe HFI was 54%, 46%, and 23%. The prevalence of severe food insecurity was 4.4 times higher when the educational level of the mother/other adult female head of household was < 6 years compared to > 10 years (see Table 2 in main paper). Compared to the food secure reference group, women from low food security (adjusted PR= 1.94; 95% C.I.=1.27, 2.96; p= 0.0001) and very low food security (adjusted PR=4.03; 95% C.I.= 2.71, 5.99; p=0.0001) were more likely than their food secure counterparts to report that their diet had been fair/poor during the past 12 months. Likewise, the mean weekly number of different food items consumed by women was reduced among those living in households with low and very low food security compared to those from food secure homes, i.e., adjusted mean=36.6 items vs. 41.2 items vs. 43.3 items (see Table 3 in main paper).

**REFERENCES**

1.Nord M. Introduction to Item Response Theory applied to Food Security Measurement: Basic Concepts, Parameters, and Statistics. Technical Paper, FAO, Rome, 2014. Accessed at: http://www.fao.org/economic/ess/ess-fs/voices/en.

2.Coleman-Jensen A, Rabbitt MP, Gregory C, Singh A. *Household Food Security in the United States in 2014*, ERR-194, U.S. Department of Agriculture, Economic Research Service, 2015.

3. Bickel G, Nord M, Price C, Hamilton W, Cook J. *Guide to Measuring Household Food Security, Revised 2000*. U.S. Department of Agriculture, Food and Nutrition Service, Alexandria VA. March, 2000.

4. Linacre JM. Winsteps® Ministep Rasch-Model Computer Programs. Program Manual 3.91.0, 2015. *Accessed* at: *http//www.* winsteps.com.

5. Linacre JM, Wright BD. Dichotomous mean-square infit and outfit Chi-square fit statistics. *Rasch Measurement Transactions* 8(2):360, 1994.

6. Coates J, Frongillo EA, Rogers BL, Webb P, Wilde PE, Houser R. [Commonalities in the experience of household food insecurity across cultures: what are measures missing?](http://www.ncbi.nlm.nih.gov/pubmed/16614441) *Journal of Nutrition* 136(5):1438S-1448S, 2006.

7. Nord M. *Measuring the food security of elderly persons. Family Economist Nutrition Reviews 15:33–45, 2003.*

8. Institute of Medicine (IOM). *Hunger and Obesity: Understanding a Food Insecurity Paradigm: Workshop Summary.* Washington, DC: The National Academies Press, 2011.

**Table 1. Response Characteristics, Item-Fit of Items, and Item Severity of the Quito HFSSM (n=794)**

| **Item Name** |  | **Affirmative responses** | **Item infit** | **Item outfit** | **Item**  **Severity^1^** | **SE** |
| --- | --- | --- | --- | --- | --- | --- |
| HH2 | Worried food would run out | 87.9 | 1.25 | 4.58 | -4.32 | 0.14 |
| HH3 | Food bought did not last | 81.7 | 1.04 | 3.43 | -3.56 | 0.12 |
| CH1 | Fed child low-cost meals | 80.5 | 0.96 | 1.99 | -3.43 | 0.12 |
| HH4 | Could not afford balanced meals | 78.6 | 1.00 | 1.67 | -3.24 | 0.12 |
| CH2 | Couldn’t feed child balanced meals | 73.5 | 0.92 | 3.80 | -2.75 | 0.11 |
| CH3 | Child not eating enough | 59.2 | 0.93 | 1.01 | -1.56 | 0.10 |
| AD2 | Adult ate less than she/he felt should | 39.1 | 0.89 | 1.02 | 0.04 | 0.11 |
| AD3 | Adult hungry but did not eat | 38.9 | 0.98 | 0.98 | 0.06 | 0.11 |
| CH4 | Cut size of child’s meals | 37.5 | 0.94 | 0.89 | 0.18 | 0.11 |
| AD1 | Adult cut or skipped meals | 29.6 | 0.85 | 0.78 | 0.88 | 0.11 |
| CH6 | Child went hungry | 27.0 | 0.90 | 0.67 | 1.15 | 0.12 |
| AD4 | Adult lost weight | 26.7 | 1.03 | 1.70 | 1.17 | 0.12 |
| AD1A | Adult cut or skipped meals, 3 or more months | 23.7 | 0.95 | 0.89 | 1.48 | 0.12 |
| CH5 | Child skipped meals | 18.1 | 0.91 | 1.35 | 2.16 | 0.13 |
| AD5 | Adult did not eat for a whole day | 16.3 | 1.03 | 2.38 | 2.41 | 0.14 |
| CH5a | Child skipped meals, 3 or more months | 14.2 | 0.91 | 2.11 | 2.74 | 0.16 |
| AD5a | Adult did not eat for a whole day, 3 or more months | 11.9 | 0.92 | 0.53 | 3.15 | 0.16 |
| CH7 | Child did not eat for a whole day | 10.4 | 1.12 | 0.77 | 3.46 | 0.17 |

**^1^** The scaling analysis did not include the 717 households that affirmed none (n=25) or all (n=52) of the items in the Quito HFSSM since those do not provide information regarding the severity of items relative to one another.

**Table 2. Differential Item Functioning (DIF) Analysis Results**

|  |  | **Low education**^1^ | | **Low household income**^2^ | | **Full-time housewife**^3^ | | **Long-term neighborhood residency**^4^ | |
| --- | --- | --- | --- | --- | --- | --- | --- | --- | --- |
| **Item Name** |  | **DIF contrast** | **Welch t** | **DIF contrast** | **Welch t** | **DIF contrast** | **Welch t** | **DIF contrast** | **Welch t** |
| HH2 | Worried food would run out | -0.20 | -0.70 | -0.40 | -0.62 | -0.18 | -0.66 | -0.20 | 0.71 |
| HH3 | Food bought did not last | -0.09 | -0.35 | 0.38 | 0.60 | 0.20 | 0.84 | -0.51 | -2.05** |
| CH1 | Fed child low-cost meals | 0.32 | 1.39 | -0.16 | -0.29 | 0.17 | 0.71 | -0.30 | -1.23 |
| HH4 | Could not afford balanced meals | 0.37 | 1.51 | 0.34 | 0.58 | 0.00 | 0.00 | 0.04 | 0.18 |
| CH1 | Fed child low-cost meals | 0.32 | 1.39 | -0.16 | -0.29 | 0.17 | 0.71 | -0.30 | -1.23 |
| CH2 | Couldn’t feed child balanced meals | -0.5 | -0.22 | -0.71 | -1.54 | 0.00 | 0.00 | -0.15 | -0.65 |
| CH3 | Child not eating enough | -0.19 | -0.90 | -0.04 | -0.08 | 0.11 | 0.54 | -0.06 | -0.27 |
| AD2 | Adult ate less than she/he felt should | -0.25 | -1.17 | 0.32 | 0.78 | 0.22 | -1.02 | 0.20 | 0.94 |
| AD3 | Adult hungry but did not eat | -0.09 | -0.40 | 0.67 | 1.65 | 0.00 | 0.00 | 0.15 | 0.69 |
| CH4 | Cut size of child’s meals | 0.57 | 2.00* | -0.36 | -0.88 | 0.00 | 0.00 | 0.31 | 1.40 |
| AD1 | Adult cut or skipped meals | -0.11 | -0.46 | -0.10 | -0.25 | 0.00 | 0.00 | 0.28 | 1.24 |
| AD1A | Adult cut or skipped meals > 3 mos. | -0.21 | -0.97 | -0.59 | -1.30 | -0.09 | -0.35 | 0.10 | 0.40 |
| CH6 | Child went hungry | 0.18 | 0.75 | 0.16 | 0.38 | 0.19 | 0.82 | 0.61 | 2.61*** |
| AD4 | Adult lost weight | -0.14 | -0.50 | 0.73 | 1.76 | 0.00 | 0.00 | 0.33 | 1.41 |
| CH5 | Child skipped meals | 0.31 | 0.99 | -0.06 | -0.13 | 0.10 | 0.38 | -0.35 | -1.29 |
| AD5 | Adult did not eat for a whole day | 0.31 | 1.22 | 0.21 | 0.44 | -0.32 | -1.12 | -0.48 | -1.68 |
| CH5a | Child skipped meals > 3 mos. | 0.31 | 0.99 | -0.62 | -1.04 | -0.14 | -0.45 | -0.35 | -1.29 |
| AD5a | Adult did not eat for a whole day > 3 mos. | 0.18 | 0.75 | -0.42 | -0.79 | -0.16 | -0.49 | -0.26 | -0.86 |
| CH7 | Child did not eat for a whole day | -0.37 | -1.04 | 0.05 | 0.09 | 0.38 | 1.05 | -01.7 | -0.49 |

*p=0.046; **p=0.04; *** p=0.009

^1^ Adult female participant education < 6 vs. > 6 years, ^2^ Household *per capita* income < $50/month vs. > $50/month, ^3^ Adult female participant occupation (full-time housewife vs. other), ^4^ Adult female participant neighborhood residency > 50% vs. < 50% of lifetime

**Figure 1. Comparison of Calibrations (severity parameter estimates) of Items in the Quito HFSSM with those in the U.S. CPS Food Security Supplement^1^**


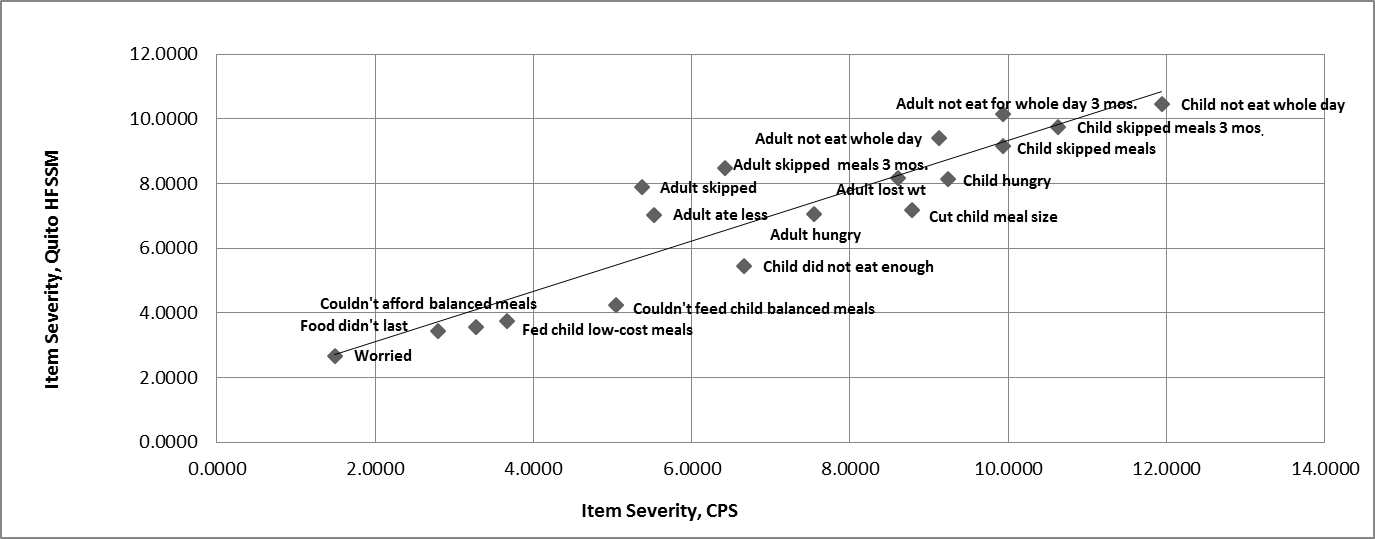


^1^ The calibrations of items from the Quito HFSSM were adjusted by a linear transformation to equate the mean and standard deviation of the items to the U.S. CPS [3]
